# Evaluation of Serum CEA, CA19-9, CA72-4, CA125 and Ferritin as Diagnostic Markers and Factors of Clinical Parameters for Colorectal Cancer

**DOI:** 10.1038/s41598-018-21048-y

**Published:** 2018-02-09

**Authors:** Yanfeng Gao, Jinping Wang, Yue Zhou, Sen Sheng, Steven Y. Qian, Xiongwei Huo

**Affiliations:** 1 Department of Anesthesiology, the First Affiliated Hospital of Xi’an Jiaotong University, Xi’an, China; 2 Department of Gynecology, the Northwest Women and Children Hospital, Xi’an, China; 3 Department of Statistics, North Dakota State University, North Dakota, 58102 USA; 4 Department of Neurosurgery, Neuroscience Institute, Baylor Scott and White Health, Temple, Texas 76502 USA; 5 Department of Pharmaceutical Sciences, North Dakota State University, North Dakota, 58105 USA; 6 Department of General Surgery, the First Affiliated Hospital of Xi’an Jiaotong University, Xi’an, China

## Abstract

Blood-based protein biomarkers have recently shown as simpler diagnostic modalities for colorectal cancer, while their association with clinical pathological characteristics is largely unknown. In this study, we not only examined the sensitivity and reliability of single/multiple serum markers for diagnosis, but also assessed their connection with pathological parameters from a total of 279 colorectal cancer patients. Our study shown that glycoprotein carcinoembryonic antigen (CEA) owns the highest sensitivity among single marker in the order of CEA > cancer antigen 72-4 (CA72-4) > cancer antigen 19-9 9 (CA19-9) > ferritin > cancer antigen 125 (CA125), while the most sensitive combined-markers for two to five were: CEA + CA72-4; CEA + CA72-4 + CA125; CEA + CA19-9 + CA72-4 + CA125; and CEA + CA19-9 + CA72-4 + CA125 + ferritin, respectively. We also demonstrated that patients who had positive preoperative serum CEA, CA19-9, or CA72-4 were more likely with lymph node invasion, positive CA125 were prone to have vascular invasion, and positive CEA or CA125 were correlated with perineural invasion. In addition, positive CA19-9, CA72-4, or CA125 was associated with poorly differentiated tumor, while CEA, CA19-9, CA72-4, CA125 levels were positively correlated with pathological tumor-node-metastasis stages. We here conclude that combined serum markers can be used to not only diagnose colorectal cancer, but also appraise the tumor status for guiding treatment, evaluation of curative effect, and prognosis of patients.

## Introduction

Colorectal cancer (CRC) is the third most common cancer in men and the second in women worldwide, which accounts for approximately 10% of all cancer deaths^1^. The survival of CRC is significantly dependent on the stage of cancer at diagnosis, with the 5 year survival rate at around 90% for localized disease, 70% for regional disease, and only 13% for distantly metastatic CRC^2^. In general, CRC is not the sudden lesions in the colorectal mucosa, but through a sequence of development as “normal mucosa - adenoma - cancer”^3,4^. Due to the slow and progressive nature of CRC, the majority of this disease has been shown as preventable and potentially curable by early detection and the removal of precancerous polyps or early stage tumors^5,6^, Therefore, early detection of CRC is crucial to reduce incidence and mortality associated with the disease.

Currently, the most widely used diagnostic approaches for CRC are the endoscopic procedures, such as colonoscopy, sigmoidoscopy, and computed tomography (CT) colonography, with high sensitivity and specificity for identifying polyps and cancers^7–9^. However, the complexity of implementation, high cost, invasiveness, time consuming procedures, as well as requirement of repeating (3–5 years), have resulted in poor compliance rates^10,11^. Some inexpensive and non-invasive methods, such as the fecal occult blood test (FOBT) based screening, have also been developed, but with lower sensitivity and specificity^12–14^. There is a great need to develop simpler, less invasive and accurate tests to improve the diagnosis of CRC.

In recent years, with the advanced knowledge of tumor mechanism and development of molecular biology technology, detection of tumor markers has been commonly used for early screening and diagnosis of cancer, guidance of cancer treatment, evaluation of curative effect, monitoring of cancer recurrence and metastasis, and the judgment of prognosis and survival^15^. Accumulative studies have reported the use of blood biomarkers as non-invasive diagnostic approach to detect CRC^16^. Currently, the glycoprotein carcinoembryonic antigen (CEA) is the most widely used blood-based CRC molecular marker that has been proved as a valuable tool for patient monitoring^17^. From the prognosis standpoint, it appears reasonable to examine CEA expression before surgery especially in patients with metastasis.

However, the major concern of using CEA as a marker for CRC is its association with other types of cancers, e.g. ovarian cancer, and the benign conditions (inflammatory bowel disease). Other serum markers such as cancer antigen 19-9 (CA19-9)^18,19^, cancer antigen 125 (CA125)^20,21^, cancer antigen 72-4 (CA72-4)^22,23^, serum ferritin (SF)^24,25^ have also been used as the indicators for CRC diagnosis and for post-operative surveillance, as well as monitoring treatment effects. Due to the highly heterogeneous nature of CRC, single tumor marker is unlikely to become a stand-alone diagnostic test as the commonly insufficient sensitivity and/or specificity. Using a panel of tumor markers for CRC diagnosis has potentially to be effective approach. Moreover, the limited knowledge of the relationship between the biomarkers and the clinical parameters of CRC has obstructed the optimized using of biomarkers on getting more accurate and meaningful information for CRC diagnosis and treatment outcomes.

Herein, we here present the analysis of the diagnostic sensitivity of single and combination of preoperative serum tumor markers, including CEA, CA125, CA19-9, CA72-4 and SF in CRC patients. The correlation between CRC pathological parameters and the evaluated markers of diagnostic test for CRC was also assessed for the first time.

## Results

### Sensitivity and Specificity assessment of individual and combinational examination of serum CEA, CA19-9, CA72-4, CA125 and SF in CRC patients

We first analyzed the sensitivity of individual marker, *e*.*g*. CEA, CA19-9, CA72-4, CA125 and SF. CEA has the highest sensitivity (46.59%) followed by CA72-4 (44.80%), while CA19-9, CA125, and SF were all lower than 15% (CA125 has the lowest sensitivity ~10.04%, Table 1). CA125 has the highest specificity (99%) of individual maker, followed by CA72-4, SF, CA19-9, while CEA has the lowest specificity (80%). Combined tests of any two of the five tumor markers, the sensitivities were in generally improved compared to single marker testing. The sensitivity range of two combination was 18.64% to 60.93%; the highest sensitivity is the combination of CEA + CA72-4 (Table 1) which was significantly lower than the sum of sensitivities (91.39%) of these two markers tested individually (46.59% + 44.80% in Table 1). This result suggests that there is a positive correlation of the two markers. In order words, many patients could be double positive to CEA and CA72-4 (Table 1).

**Table 1:** The sensitivity of individual and combinational examination of serum biomarkers in CRC patients.

	Markers	Sensitivity	Specificity
Single Marker	CEA	46.59% (130/279)	80% (59/74)
	CA199	14.39% (41/279)	89% (66/74)
	CA724	44.80% (125/279)	97% (72/74)
	CA125	10.04% (28/279)	99% (73/74)
	SF	10.39% (29/279)	95% (70/74)
Two Markers Combination	CEA + CA199	49.46% (138/279)	78% (58/74)
	CEA + CA724	60.93% (170/279)	77% (57/74)
	CEA + CA125	53.05% (148/279)	78% (58/74)
	CEA + SF	52.69% (147/279)	77% (57/74)
	CA199 + CA724	52.33% (146/279)	86% (64/74)
	CA199 + CA125	21.86% (61/279)	89% (66/74)
	CA199 + SF	21.51% (60/279)	85% (63/74)
	CA724 + CA125	51.25% (143/279)	96% (71/74)
	CA724 + SF	51.97% (145/279)	92% (68/74)
	CA125 + SF	18.64% (52/279)	93% (69/74)
Three Markers Combination	CEA + CA199 + CA724	63.08% (176/279)	76% (56/74)
	CEA + CA199 + CA125	54.12% (151/279)	78% (58/74)
	CEA + CA199 + SF	53.41% (149/279)	76% (56/74)
	CEA + CA724 + CA125	65.59% (183/279)	76% (56/74)
	CEA + CA724 + SF	64.87% (181/279)	74% (55/74)
	CEA + CA125 + SF	57.71% (161/279)	76% (56/74)
	CA199 + CA724 + CA125	56.99% (159/279)	86% (64/74)
	CA199 + CA724 + SF	55.56% (155/279)	82% (61/74)
	CA199 + CA125 + SF	27.24% (76/279)	85% (63/74)
	CA724 + CA125 + SF	56.99% (159/279)	91% (67/74)
Four to Five Markers Combination	CEA + CA199 + CA724 + CA125	66.67% (186/279)	76% (56/74)
	CEA + CA199 + CA724 + SF	65.59% (183/279)	73% (54/74)
	CEA + CA199 + CA125 + SF	56.63% (158/279)	76% (56/74)
	CEA + CA724 + CA125 + SF	66.31% (185/279)	73% (54/74)
	CA199 + CA724 + CA15 + SF	60.22% (168/279)	82% (61/74)
	CEA + CA199 + CA724 + CA125 + SF	67.38% (188/279)	73% (54/74)

The detection sensitivities were further increased when three biomarkers are combined at range of 27.24% (CA19-9 + CA125 + SF) to 65.59% (CEA + CA72-4 + CA125) (Table 1). When four markers combined, the sensitivity range was further increased from 56.63% (CEA + CA19-9 + CA125 + SF) to 66.67% (CEA + CA19-9 + CA72-4 + CA125), while sensitivity of all five tumor markers combination reached 67.38% (Table 1). Thus, the highest sensitivity of single and combined markers from two to five were: CEA, CEA + CA72-4, CEA + CA72-4 + CA125, CEA + CA19-9 + CA72-4 + CA125, and CEA + CA19-9 + CA72-4 + CA125 + SF, respectively. There were significant differences for any of these combined groups vs. CEA detection alone (p < 0.05). However, no significant differences when comparing CEA + CA72-4 with the combinations of three, four or five markers (p > 0.05). In comparison, with the increase of sensitivity by combining the tumor markers, there is a slight decrease in specificity. The highest specificity of single and combined markers from two to five were: CA125, CA72-4 + CA125, CA72-4 + CA125 + SF, CA19-9 + CA72-4 + CA125 + SF, and CEA + CA19-9 + CA72-4 + CA125 + SF, respectively (Table 1).

### Biomarkers vs. clinicopathological parameters of CRC

In order to determine whether these biomarkers are associated with pathological parameters of CRC, we further assessed corrections between CRC pathological parameters (i.e., lymph node metastasis, vascular invasion, etc.) with CEA, CA19-9, CA72-4, CA125 and SF (Table 2) as following:

1. Chi-square test results showed that the preoperative serum CEA expression was significantly different in the presence or absence of lymph node metastasis, nerve infiltration, or pathological tumor-node-metastasis (pTNM) staging (p < 0.05). In contrast, there was no significant difference for patients with or without vascular invasion and tumor differentiation, or with different sex (p > 0.05);
2. The relationship of CA19-9 with the clinical and pathological parameters of colorectal cancer was also analyzed. There were significant statistical differences for preoperative serum CA19-9 expression in the presence or absence of lymph node metastasis, tumor differentiation, or pTNM staging (p < 0.05). However, there was no significant difference of CA19-9 expression for patients with different vascular invasion, nerve invasion, and sex statuses (p > 0.05);
3. Similar to CA19-9, the expression of serum CA72-4 had statistical significance in patients with or without lymph node metastasis, tumor differentiation, or pTNM staging (p < 0.05). In addition, according to our study, patient age didn’t influence the serum levels of CEA, CA19-9, CA125 and SF, but showed statistical significant difference for CA72-4 levels in patients younger than 60 vs. older than 60 (p < 0.05). However, there was no significant difference for patients with different vascular infiltration, nerve invasion, and sex statuses (p > 0.05);
4. Similarly, there were statistically significant differences for preoperative serum CA125 expression in the presence or absence of lymph node metastasis, tumor differentiation, or pTNM staging (p < 0.05). While there was no significant difference in CA125 sensitivity for patients with different vascular infiltration, nerve invasion and sex statuses (p > 0.05); and
5. In contrary to all other markers, no significant difference was observed for preoperative serum SF expression in the presence or absence of lymph node metastasis, vascular invasion, nerve invasion, tumor differentiation, or pTNM staging, as well as for different gender (p > 0.05).

**Table 2:** The relationship of biomarkers and clinicopathological parameters of CRC.

Clinicopathological parameters	Cases	CEA				CA199				CA724				CA125				SF			
		Pos	Neg	*p*	***Χ*** ^***2***^	Pos	Neg	*p*	***Χ*** ^**2**^	Pos	Neg	*p*	***Χ*** ^***2***^	Pos	Neg	*p*	***Χ*** ^***2***^	Pos	Neg	*p*	***Χ*** ^***2***^
**Age**																					
<60	106	46	60	0.402	0.703	14	92	0.583	0.302	62	44	0	12.951	8	98	0.279	1.173	14	92	0.228	1.453
≥60	173	84	89			27	146			63	110			20	153			15	158		
**Gender**																					
Male	157	78	79	0.241	1.375	20	137	0.295	1.096	72	85	0.687	0.162	11	146	0.056	3.65	15	142	0.602	0.272
**Female**	122	52	70			21	101			53	69			17	105			14	108		
**Lymphatic metastasis**																					
Yes	103	59	44	0.006	7.493	24	79	0.002	9.646	65	38	0	22.121	8	95	0.335	0.931	10	93	0.774	0.082
No	176	71	105			17	159			60	116			20	156			19	157		
**Vascular invasion**																					
Yes	18	9	9	0.765	0.09	4	14	0.351	0.87	10	8	0.343	0.9	6	12	0	21.93	2^*^	16	1	0.011
No	261	121	140			37	224			115	146			22	239			27	234		
**Nerve infiltration**																					
Yes	15	12	3	0.008	7.109	0^*^	15	0.138	2.731	7	8	0.881	0.022	5	10	0.01	9.53	3^*^	12	0.195	1.57
No	264	118	146			41	223			118	146			23	241			26	238		
**Degree of differentiation**																					
Poorly	35	18	17	0.401	1.828	11	24	0.007	10.004	7	28	0.006	10.35	4	31	0.029	7.097	2^*^	33	0.619	0.959
Moderately	236	110	126			28	208			115	121			21	215			26	210		
Highly	8	2^*^	6			2^*^	6			3^*^	5			3^*^	5			1^*^	7		
**pTNM staging**																					
Stage I	51	12	39	0	24.604	1	50	0	50.275	10	41	0	28.329	2^*^	49	0.011	6.479	6	45	0.783	1.075
Stage II	117	51	66			12	105			49	68			9	108			14	103		
Stage III	96	54	42			17	79			53	43			14	82			8	88		
Stage IV	15	13	2			11	4			13	2			3*	12			1*	14		

*Indicates that the theoretical frequency of the four-cell table is less than 5, and the statistical analysis was performed using Fisher exact probability method. Pos: Positive; Neg: Negative.

### Binary logistic regression of biomarkers vs. CRC clinicopathological parameters

To further understand the relationship between the biomarkers with colorectal cancer clinic-pathological parameters, binary logistic regression analysis were also performed. Our study indicated that lymph node metastasis, vascular invasion, nerve infiltration and TNM staging can significantly affect CEA level in CRC patients (Table 3). In addition, the levels of CA19-9, CA72-4, and CA125 were also influenced by lymph node metastasis, tumor differentiation and TNM staging. However, SF levels were not affected by the presence or absence of lymph node metastasis, vascular invasion, nerve invasion, tumor differentiation, as well as the pTNM staging.

**Table 3:** Binary logistic regression analysis of biomarkers and CRC clinicopathological parameters.

Markers	Clinicopathological parameters	B	SE	Wald	df	p	Exp (B)
CEA	Age	−0.208	0.248	0.702	1	0.402	0.812
	Gender	0.317	0.292	1.18	1	0.277	1.374
	Lymphatic Metastasis	2.216	0.392	31.878	1	0	9.17
	Vascular Invasion	−1.495	0.768	9.419	1	0.011	0.143
	Nerve Infiltration	4.407	0.815	29.253	1	0	82.044
	Differentiation Degree	0.047	0.388	0.014	1	0.904	1.048
	pTNM Staging	−1.887	0.26	52.526	1	0	0.152
CA19-9	Age	−0.195	0.355	0.301	1	0.583	0.823
	Gender	−0.522	0.402	1.681	1	0.195	0.594
	Lymphatic Metastasis	1.819	0.431	17.785	1	0	6.163
	Vascular Invasion	19.558	16907	0	1	0.999	3.12E + 08
	Nerve Infiltration	−37.9	19722	0	1	0.998	0
	Differentiation Degree	0.911	0.489	3.463	1	0.043	2.486
	pTNM Staging	−1.335	0.278	23.057	1	0	0.263
CA72-4	Age	0.900	0.253	12.700	1	0.000	2.460
	Gender	0.515	0.622	0.685	1	0.408	1.673
	Lymphatic Metastasis	0.988	0.285	12.034	1	0.001	2.685
	Vascular Invasion	22.359	23086	0	1	0.999	5.13E + 09
	Nerve Infiltration	−21.18	23086	0	1	0.999	0
	Differentiation Degree	0.596	0.274	4.714	1	0.03	0.551
	pTNM Staging	−1.193	0.244	24.007	1	0	0.303
CA125	Age	−0.471	0.438	1.156	1	0.282	0.624
	Gender	−0.14	0.521	0.073	1	0.788	0.869
	Lymphatic Metastasis	0.079	0.806	0.01	1	0.922	1.082
	Vascular Invasion	1.156	0.758	2.325	1	0.027	3.178
	Nerve Infiltration	2.427	0.985	6.072	1	0.014	11.327
	Differentiation Degree	−0.981	0.444	4.871	1	0.027	0.375
	pTNM Staging	−1.085	0.388	7.842	1	0.005	0.338
SF	Age	0.472	0.394	1.433	1	0.231	1.603
	Gender	−0.273	0.403	0.459	1	0.98	0.761
	Lymphatic Metastasis	−0.83	0.609	1.857	1	0.173	0.436
	Vascular Invasion	−1.045	1.235	0.715	1	0.398	0.352
	Nerve Infiltration	1.435	1.155	1.545	1	0.214	4.201
	Differentiation Degree	−0.4	0.53	0.568	1	0.451	0.67
	pTNM Staging	0.474	0.368	1.655	1	0.198	1.606

### The correlation of the preoperative expression of serum tumor markers with pTNM staging in CRC patients

**Figure 1:**
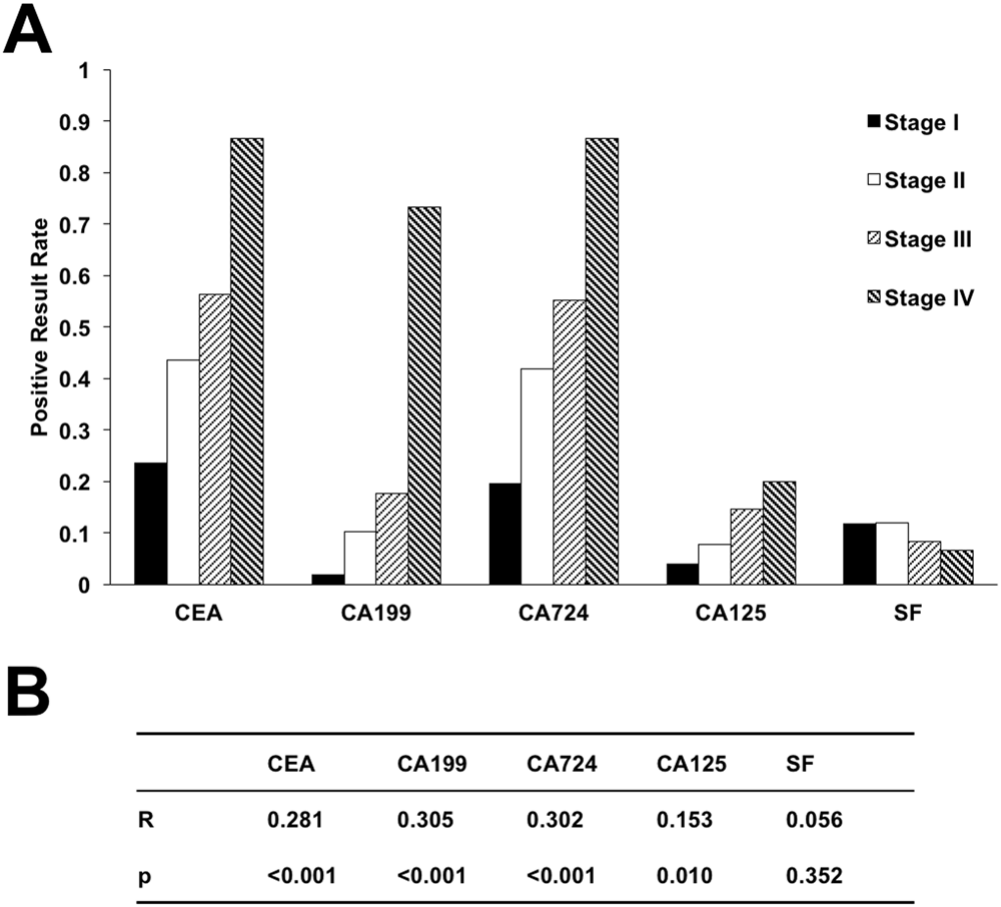
The correlation of the preoperative expression of serum tumor markers with pTNM staging. (**A**) The sensitivities of biomarkers in patients at different pTNM stages. (**B**) The correlation between preoperative serum tumor markers and the pTNM staging.

To confirm pTNM staging is correlated with the most of tested biomarkers (Table 2), we also assessed whether the markers can also reflect the stages of colorectal cancer form comparing their sensitivity *vs*. pTNM stage, *e*.*g*. analyzing the correlation between preoperative serum tumor markers and the pTNM staging. The CEA, CA19-9, CA72-4 and CA125 demonstrated a positive correlation with the advancement of tumor stages (Fig. 1A). There was also a correlation between preoperative serum tumor marker expression and the pTNM staging of CRC (Fig. 1B). As expected, CEA, CA19-9, CA72-4, CA125 were positively correlated with tumor stages (p < 0.05), while SF had no correlation with tumor stage.

## Discussion

Identification of molecular markers could improve the diagnosis and facilitate disease management in CRC. Especially, the use of serum protein biomarkers holds the promise for the development of effective, non-invasive, and inexpensive tests for the detection of CRC. For the first time, we not only measured the preoperative levels of five serum tumor markers, CEA, CA19-9, CA72-4, CA125 and SF, in a total of 279 CRC patients, but also analyzed the correlation of these markers with the pathological parameters of CRC. Our data show that combined tumor markers improves the diagnostic sensitivity using single marker. Although there was no significant difference on the highest sensitivities among two, three, four or five, the combined CEA + CA724 detection is the best combination to achieve sensitive and cost-efficient results. In addition, our study also demonstrated that lymphatic metastasis, vascular invasion, nerve infiltration, tumor differentiation, as well as pTNM stage, all can be the factor to influence the levels of tumor markers. We thus proposed that levels of CEA, CA19-9, CA72-4, and CA125 can be used as the biomarkers to not only diagnose CRC, but also predict CRC lymph node metastasis, vascular invasion, neural infiltration, tumor differentiation and staging, therefore to be used to guide the treatment and prognosis of CRC.

CEA has shown great value for the differential diagnosis of malignant tumors, disease monitoring and evaluation of efficacy, especially for postoperative monitoring of CRC. The sensitivity of CEA in our study was 46.59% (130/279, Table 2) consistent with previous reports^26^. Preoperative serum CEA positive and negative expressing patients have significant different conditions of lymph node metastasis, nerve infiltration and pTNM staging. However, there was no significant difference in vascular invasion and tumor differentiation, indicating that CEA may be implicated in CRC metastasis and nerve infiltration. The positive rates of CEA in stage I through stage IV were 24%, 44%, 56% and 87%, respectively (Table 3). The spearman correlation coefficient between CEA and staging was 0.281 (p < 0.05), suggesting that there is a weak positive linear correlation between CEA and stages of pTNM.

At the same time, regression analysis showed that lymph node metastasis, vascular invasion, nerve infiltration and pTNM staging are the factors that affect CEA level. Different results we observed for the relationship between CEA and vascular invasion are most likely due to the relatively small sample size in the patients with vascular invasion (n = 18). However, our study also suggested that with larger trials to standardize the correlation of CEA with the clinicopathological parameters of CRC, it is possible to measure serum CEA levels for predicting CRC disease status, e.g., tumor staging and lymph node metastasis, and also providing the guides for better clinical treatment outcome and prognosis.

CA72-4 is currently one of the primary markers for the diagnosis of gastric cancer and monitoring the course and efficacy of gastric cancer^27,28^. The sensitivity of CA72-4 is 65-70%, significantly higher than that of CA19-9 and CEA^29^. In ovarian cancer, it has a higher sensitivity for mucin-type ovarian cancer, and its combination with CA125 for detection can significantly improve the clinical sensitivity^30^. In CRC, CA72-4 combined with CEA detection was also found to significantly improve the sensitivity of the initial diagnosis^31^. Compared to CA19-9 and CEA, the most important advantage of CA72-4 is its high clinical specificity for detecting benign lesions^32^. However, it is still lack of reliable evidence for the current application of individual use of CA72-4 serum for testing CRC benign and malignant prognosis, tumor staging, monitoring for recurrence and/or metastasis. In our study, the sensitivity of CA72-4 in CRC was 44.80% (125/279, Table 2), consistent with the Park *et al*.’s study^33^. The level of serum CA72-4 was closely related to lymph node metastasis, tumor differentiation and pTNM staging, but not to nerve infiltration and vascular invasion. Noteworthy, the joint detection of CEA + CA72-4 was with the highest sensitivity among the two marker combinations, and there was no significant difference between CEA + CA72-4 and three, four and five markers combination (p > 0.05). Our study suggested that the combination of CEA and CA72-4 can be used to achieve better diagnostic sensitivity for CRC than using CEA individual.

Besides the cancer antigens, we also analyzed the levels of ferritin, a large molecular weight iron-containing protein in CRC serum. A large number of clinical and epidemiological studies have confirmed that serum iron levels were significantly increased in a variety of solid tumors patients, which may be due to the increased synthesis of SF by tumor cells or the release of SF from damaged tissue^34^. With the continuous study of the relationship between ferritin and disease, more studies have shown that abnormal ferritin levels are associated with many diseases (such as cardiovascular and cerebrovascular diseases and type 2 diabetes, etc.), indicating that ferritin can be used as adjuncts in these diseases diagnose and therap^35,36^. In our study, however, the sensitivity of SF in CRC was only 10.39% (29/279, Table 2). Although SF showed no statistically significant correlation with lymph node metastasis, vascular invasion, nerve infiltration, tumor differentiation and pTNM staging, the combined biomarkers with SF indeed showed improved sensitivity for diagnosis of CRC (Table 1). The mechanism of elevated levels of SF in CRC and the use of SF as an adjunct diagnosis indicator for CRC requires further investigation.

Since effective screening and early diagnosis are the main measures to reduce the incidence and mortality of CRC, more mechanistic studies and well-designed trials of studying promising new candidate biomarkers and new combinations to improve diagnostic performance will be valuable for the management of CRC. By analyzing five serum tumor markers from 279 patents, we concluded that significantly improved sensitivity for CRC diagnosis can be reached by using combined markers rather than using CEA alone. For the first time, we are able to establish the correlation between these makers with the clinic-pathological status of CRC. The after-operation follow-up evaluation of these biomarkers would definitely generate valuable information for the understanding and management of CRC, which awaits future investigation. To warrant future investigations for the functional relationship of CRC biomarkers and the clinic-pathological status of CRC, as well as the underlying biological mechanisms, we here have provided a valuable procedure to facilitate the diagnosis, prognosis, and therapeutic management of CRC.

## Methods

The human subject studies were approved by the ethical standards committee of Xi’an Jiaotong University. All experiments were performed in accordance with relevant guidelines and regulations of Xi’an Jiaotong University. Written informed consents were obtained from the patients participating in the study. Preoperative serum tumor marker test was analyzed using the serum samples from a total of 279 CRC patients, including 157 male patients, 122 female patients, aged 22 to 85 years (mean 62.1 ± 11.9 years). To assess the specificity of tested markers, 74 non-CRC individuals were included, including 35 male, and 39 female, aged 32 to 84 years (mean 60.45 ± 12.4 years). According to the TNM staging description (seventh edition) of the International Union Against Cancer (UICC)/American Cancer Society (AJCC), the stages of colorectal cancer in patients based on the results of postoperative pathological examination include: 51 patients were with stage I, 117 patients were with stage II, 96 patients were with stage III, 15 patients were with stage IV CRC (Table 4**)**.

**Table 4:** Clinical characters of CRC patients recruited in this study.

Group	Case	Percentage
**Gender**		
Male	157	56.27%
Female	122	43.73%
**Age**		
<60	106	37.99%
≥60	173	62.01%
**Tumor Location**		
Colon	78	27.96%
Rectum	201	72.04%
**Tumor Type**		
Ulcerative adenocarcinoma	191	68.46%
Uplift type adenocarcinoma	56	20.07%
Ulcerative mucinous adenocarcinoma	4	1.43%
Uplift mucinous adenocarcinoma	3	1.08%
Diffuse adenocarcinoma	1	0.36%
Invasive adenocarcinoma	2	0.72%
Tan umbrella-shaped adenocarcinoma	1	0.36%
Tubular adenoma carcinogenesis	15	5.38
Other	6	2.15%
**Degree of Differentiation**		
Poorly differentiation	35	12.54%
Moderately differentiation	236	84.59%
Highly differentiation	8	2.87%
**Lymph Node Metastasis**		
Yes	103	36.92%
No	176	63.08%
**Vascular Invasion**		
Yes	18	6.45%
No	261	93.55%
**Nerve Infiltration**		
Yes	15	5.38%
No	264	94.62%
**pTNM Staging**		
Stage I	51	18.28%
Stage II	117	41.94%
Stage III	96	34.41%
Stage IV	15	5.38%

Patients with CRC who were hospitalized from Jan. 2014 to Dec. 2015 in the First Affiliated Hospital of Xi’an Jiaotong University were screened with inclusion criteria:

1. All patients who were included in this study had surgery: Patients at stage I, II, III cancer were with radical surgery, while patients at stage IV were with palliative surgery Postoperative pathological examinations were performed to confirm the colorectal cancer in all patients;
2. Patients did not receive preoperative radiotherapy or chemotherapy or other tumor-related treatment;
3. All patients have complete clinical, pathological, and follow-up data.

Note, patients with no surgery or short-term patients died after surgery were not included. The patients were strictly forbidden for smoke or alcohol after admission into hospital.

Fasting venous blood was taken at 6 -7 am on the 2nd day after admission, and then subjected for quantitative analysis of the markers in the Department of Laboratory in the First Affiliated Hospital of Xi’an Jiaotong University. All lab tests, including blood collection, handling, and quantitative analysis, were performed in accordance to the standard operating procedures and the experiments were performed on a daily basis because that the reports were used to guide clinical decisions by physicians. Quantitative analysis of the markers with enzyme-linked immunosorbent assay (ELISA) were performed by following manufacturer’s instructions. The ELISA kits used were: CEA Human ELISA Kit (Thermo Fisher), Human Carbohydrate Antigen 19-9 ELISA Kit (Sigma), Human CA72-4 ELISA (Sigma), CA125 Human ELISA Kit (Thermo Fisher), and Human Ferritin ELISA Kit (Sigma). The normal reference values of the five serum tumor markers were CEA 0-3.40 ng/ml, CA19-9 0-39.0 U/ml, CA72-4 0-9.8 U/ml, CA125 0-35 U/ml, SF 30-400 U/ml. Exceeding the upper limit of the normal threshold is considered positive. For joint detection of two to five markers among CEA, CA19-9, CA72-4, CA125 and SF for sensitivity, detection of any positive tumor marker was considered the positive result. Sensitivity = number of positive cases/total number of CRC cases, while Specificity = number of negative/total number of healthy individuals.

### Statistics

All data were processed by SPSS22.0 statistical software. The statistical methods were selected by χ^2^ test, Fisher exact probability method (minimum theoretical frequency < 5), and Spearman correlation analysis. The influencing factors of tumor marker levels were analyzed by binary logistic regression analysis. Test level α = 0.05, p < 0.05 was statistically significant.
